# Computational Investigation of Contributions from Different Subtypes of Interneurons in Prefrontal Cortex for Information Maintenance

**DOI:** 10.1038/s41598-020-61647-2

**Published:** 2020-03-13

**Authors:** Qian Zhang, Yi Zeng, Taoyi Yang

**Affiliations:** 1 Research Center for Brain-inspired Intelligence, Institute of Automation, Chinese Academy of Sciences, Beijing, 100190 China; 2 University of Chinese Academy of Sciences, Beijing, 100049 China; 3 Center for Excellence in Brain Science and Intelligence Technology, Chinese Academy of Sciences, Shanghai, 200031 China; 4 National Laboratory of Pattern Recognition, Institute of Automation, Chinese Academy of Sciences, Beijing, 100190 China

**Keywords:** Neuroscience, Computational neuroscience, Network models

## Abstract

Interneurons play crucial roles in neocortex associated with high-level cognitive functions; however, the specific division of labor is still under investigation. Interneurons are exceptionally diverse in their morphological appearance and functional properties. In this study, we modify a prefrontal multicolumn circuit in which five subtypes of inhibitory interneurons play distinct roles in the maintenance of transient information. These interneurons are classified according to the extending range of axonal projections. Our work simplifies the division of labor between different types of interneurons for the maintenance of information and the principle of functional redundancy of the brain from the perspective of computational modeling. This model presents a framework to understand the cooperation between different interneurons in a recurrent cortical circuit.

## Introduction

Most of the neocortical neurons (70–80%) are excitatory neurons, and the remaining are mostly inhibitory interneurons^1,2^. Although the proportion is limited, synaptic inhibition plays a crucial role in supporting cognitive function of the cortical cortex^3,4^. Recent years have witnessed a dramatic accumulation of our knowledge about the diverse morphological, physiological, molecular, and synaptic characteristics of interneurons^5–8^. A mechanistic understanding of these interneurons and how they support cognition remains to be further demonstrated.

The computational models can integrate different levels of neuroscience data, helping us to explore the unknown. We present here, a multicolumn prefrontal cortex (PFC) circuit to elucidate distinct operations carried out by diverse interneurons. We have modified the model of a detailed data-driven single prefrontal column proposed by Durstewitz and colleagues [ModelDB (http://senselab.med.yale.edu/ModelDB/)]^9^, and used the adaptive exponential integrate-and-fire (aEIF) neuron model. The sources of anatomical structure include ferret, rodent, and primate PFC experiment^10–12^. The neuron parameters in this PFC network are derived from the experimental literature related to PFC and other areas of the neocortex^8,9,13^. Therefore, this model is a PFC network based entirely on biological experimental data.

The axons of interneurons usually arborize within a cortical column and can project laterally across columns^14,15^. Thus, our network incorporates five interneuron subtypes, according to the extending range of axonal projections. We explore the division of labor between different types of interneurons for information maintenance and prove that cortex network is modular^16,17^. It contains connector hubs that have connections distributed diversely across communities^18,19^. This structure can compensate for the loss of function when partial short-range connected interneurons are damaged.

Understanding the circuit mechanisms of signal transmission and information maintenance associated is expected to increase our understanding of high-level cognitive functions, such as working memory. The computational models help in linking the biochemical and anatomical properties to cognitive functions and predict key properties from microlevel to macrolevel.

## Materials and Methods

### Neuron model

Single neuron was simulated using the aEIF model. The voltage *V* and the adaptation variable *w* are expressed using the following two-dimensional differential equations:

1 $${{\bf{C}}}_{{\bf{m}}}\frac{{\bf{d}}{\bf{V}}}{{\bf{d}}{\bf{t}}}=-{{\bf{g}}}_{{\bf{L}}}({\bf{V}}-{{\bf{E}}}_{{\bf{L}}})s+{{\bf{g}}}_{{\bf{L}}}\exp \left(\frac{{\bf{V}}-{{\bf{V}}}_{{\bf{t}}{\bf{h}}}}{{{\boldsymbol{\Delta }}}_{{\bf{T}}}}\right)+{\bf{I}}-{\bf{w}}$$

2 $${{\boldsymbol{\tau }}}_{{\bf{w}}}\frac{{\bf{d}}{\bf{w}}}{{\bf{d}}{\bf{t}}}={\bf{a}}({\bf{V}}-{{\bf{E}}}_{{\bf{L}}})-{\bf{w}}$$

$${\bf{I}}{\bf{f}}\,{\bf{V}} > {{\bf{V}}}_{{\bf{t}}{\bf{h}}},{\bf{V}}\to {{\bf{V}}}_{{\bf{r}}},{\bf{w}}\to {\bf{w}}+{\bf{b}}$$

*C*_*m*_ is the membrane capacitance, *g*_*L*_ is the leak conductance, *E*_*L*_ is the leak reversal potential, *V*_*th*_ is the spike threshold, Δ_T_ is the slope factor, *τ*_*w*_ is the adaptation time constant, *a* is the subthreshold adaptation, and *b* is the spike-triggered adaptation. Neuron parameters are presented in Table S1.

### Synaptic properties

Neurons are connected through three types of synapses (AMPA, GABA_A_, and NMDA):

3 $${{\bf{I}}}_{{\bf{X}}}={{\bf{g}}}_{{\bf{X}}}^{{\bf{m}}{\bf{a}}{\bf{x}}}{\bf{s}}({\bf{V}})\sum _{{{\bf{t}}}_{{\bf{s}}{\bf{p}}}}{\bf{a}}({{\bf{t}}}_{{\bf{s}}{\bf{p}}})({{\bf{e}}}^{-\frac{{\bf{t}}-{{\bf{t}}}_{{\bf{s}}{\bf{p}}}-{{\boldsymbol{\tau }}}_{{\bf{D}}}}{{{\boldsymbol{\tau }}}_{{\bf{o}}{\bf{f}}{\bf{f}}}^{{\bf{X}}}}}-{{\bf{e}}}^{-\frac{{\bf{t}}-{{\bf{t}}}_{{\bf{s}}{\bf{p}}}-{{\boldsymbol{\tau }}}_{{\bf{D}}}}{{{\boldsymbol{\tau }}}_{{\bf{o}}{\bf{n}}}^{{\bf{X}}}}})({\bf{V}}-{{\bf{E}}}_{{\bf{r}}{\bf{e}}{\bf{v}}}^{{\bf{X}}}),$$

where

4 $$\begin{array}{c}{\bf{s}}({\bf{V}})=\{\begin{array}{ll}{\bf{1}}.{\bf{08}}{(1{\boldsymbol{+}}0.{\bf{19}}{\bf{e}}{\bf{x}}{\bf{p}}(-{\bf{0}}.064{\bf{V}}))}^{-1} & {\bf{f}}{\bf{o}}{\bf{r}}\,{\bf{X}}={\bf{N}}{\bf{M}}{\bf{D}}{\bf{A}}\\ 1 & {\bf{o}}{\bf{t}}{\bf{h}}{\bf{e}}{\bf{r}}{\bf{w}}{\bf{i}}{\bf{s}}{\bf{e}}\end{array}\\ {\bf{X}}\in \{{\bf{A}}{\bf{M}}{\bf{P}}{\bf{A}},\,{\bf{G}}{\bf{A}}{\bf{B}}{{\bf{A}}}_{{\bf{A}}},\,{\bf{N}}{\bf{M}}{\bf{D}}{\bf{A}}\}\end{array}$$

*E*_*rev*_ is the reversal potential, and *τ*_*off*_ and *τ*_*on*_ are the onset and offset time constants. The parameters are presented in Table S2.

Synapses were also equipped with short-term plasticity (STP) dynamics implemented in the Tsodyks and Markram model^20^.

5 $${{\bf{a}}}_{{\bf{n}}}={{\bf{u}}}_{{\bf{n}}}{{\bf{R}}}_{{\bf{n}}}$$

6 $${{\bf{u}}}_{{\bf{n}}+1}={{\bf{u}}}_{{\bf{n}}}\exp \left(\frac{-\Delta {\bf{t}}}{{{\boldsymbol{\tau }}}_{{\bf{f}}{\bf{a}}{\bf{c}}{\bf{i}}{\bf{l}}}}\right)+{\bf{U}}\left(1-{{\bf{u}}}_{{\bf{n}}}\exp \left(\frac{-\Delta {\bf{t}}}{{{\boldsymbol{\tau }}}_{{\bf{f}}{\bf{a}}{\bf{c}}{\bf{i}}{\bf{l}}}}\right)\right)$$

7 $${{\bf{R}}}_{{\bf{n}}+1}={{\bf{R}}}_{{\bf{n}}}(1-{{\bf{u}}}_{{\bf{n}}+1})\exp \left(\frac{-\Delta {\bf{t}}}{{{\boldsymbol{\tau }}}_{{\bf{r}}{\bf{e}}{\bf{c}}}}\right)+1-\exp \left(\frac{-\Delta {\bf{t}}}{{{\boldsymbol{\tau }}}_{{\bf{r}}{\bf{e}}{\bf{c}}}}\right)$$

8 $${{\bf{R}}}_{1}=1-{\bf{U}}$$

*a*_*n*_ is the relative efficiency, *u*_*n*_ is the utilization of synaptic efficacy with initial conditions *u*1 = *U* and *R*1 = 1, *τ*_*rec*_ is the recovery from depression on time, and *τ*_*facil*_ is the facilitation dominant on time. The values of *U*, *τ*_*facil*_, and *τ*_*rec*_ are 0.25, 500 ms, and 200 ms, respectively.

### Neural network model

Anatomically, the network is divided into two laminar components, representing the supragranular layers 2/3(L2/3) and infragranular layer 5(L5). The supragranular neurons are divided into six subtypes: pyramidal cells (PCs)^13,21^ and local-layer connection interneurons (LL-INs) with projections within the same layer as chandelier cells (ChCs)^22^, cross-layer connection interneurons (CL-INs) include bipolar cells (BPCs) as CL-IN-a and double banquet cells (DBCs) as CL-IN-b, which have vertically extended axonal clusters largely within a column^8^, and long-range connection interneurons (LR-INs) include large basket cells (LBCs) as LR-IN-a and Martinotti cells (MCs) as LR-IN-b^13^. These LBCs and MCs have large clusters of axons that extend not only across the cross-layers but also cross-multiple columns. The LBCs have similar electrophysiological properties to the PCs, so their neuronal parameters are the same as those of pyramidal cells in the respective layers^21^. Compared to the supragranular layer, there are four types of neurons in the infragranular layer, lacking BPCs^9,13^ (Neuron parameters are presented in Table S1).

**Figure 1:**
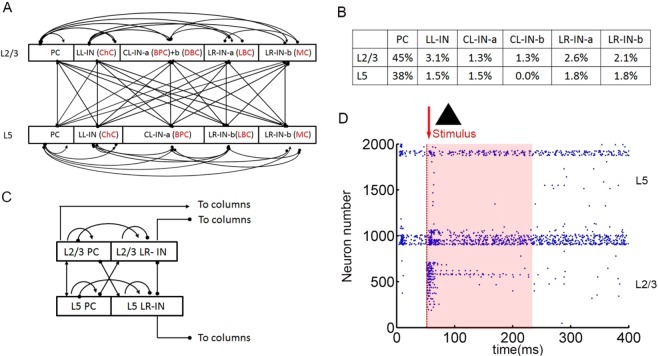
Anatomical and stimulation diagram. (**A**) Laminar structure of a single column. Arrows indicate excitatory connections and dots indicate inhibitory connections of pyramid cell (PC), local-layer connection interneuron (LL-IN), cross-layer connection interneuron (CL-IN), long-range connection interneuron (LR-IN), chandelier cell (ChC), bipolar cell (BPC), double-bouquet cell (DBC), large basket cell (LBC), and Martinotti cell (MC). (**B**) Parameters of different types of neurons are proportionally distributed in layers. (**C**) Laminar structure of multicolumn network. (**D**) Network stimulation diagram. Red arrow indicates that triangular binary image stars stimulate the L2/3 pyramidal cell. The shades of light red indicate the formation and maintenance of information.

Neurons are assumed to be organized in a single column (Fig. 1A), and each column contains 2000 neurons. The pyramidal cells and interneurons are proportionally distributed (Fig. 1B)^23,24^ and randomly connected to different connection probabilities for each pair of cell type based on previous studies^9,25,26^ (Table S3). All neurons receive background currents, which represent synaptic connections from outside the network, both within and outside the column. The excitatory neuron background current is 250 pA, and the interneuron background current is 200 pA. The connection between the columns mainly depends on PC and LR-IN, as shown in Fig. 1C.

### Stimulation paradigm

We convert the binary input image into 30 × 30 matrix of 0 s and 1 s. 0 represents no spike input and 1 represents a spike input. This matrix corresponds to 900 PC on L2/3.

- 1 column, 1 time stimulation:
  During 51~70 ms, the corresponding 1000 Hz stimulus is given to the L2/3 PC. In next 160 ms, the L2/3 PC spiking is recorded.
- 1 column, 5 times stimulation:
  During 51~70 ms, 301~320 ms, 551~570 ms, 801~820 ms, and 1051~1070 ms, the corresponding 1000 Hz stimulus is given to the L2/3 PC. In next 160 ms, the L2/3 PC spiking is recorded.
- 2–4 columns, 5 times stimulation:

Step (b) is repeated to add stimulation to every column’s L2/3 PC. In next 160 ms, the L2/3 PC spiking is recorded.

0 represents no spiking activity. When spiking occurs, it is represented by 1. Finally, the (0, 1) matrix is converted into a binary image for output. Every experiment is repeated 15 times. The performance of the original PFC network under stimulation is taken as control.

The accuracy is calculated using the following equation:

9 $${{\bf{p}}}_{{\bf{a}}{\bf{c}}{\bf{c}}{\bf{u}}{\bf{r}}{\bf{a}}{\bf{c}}{\bf{y}}}=\frac{{{\bf{N}}}_{{\bf{i}}{\bf{n}}{\bf{p}}{\bf{u}}{\bf{t}}={\bf{o}}{\bf{u}}{\bf{t}}{\bf{p}}{\bf{u}}{\bf{t}}}}{{{\bf{N}}}_{{\bf{L}}2/3{\bf{P}}{\bf{C}}}}\times 100$$

*N*_input=output_ is the number of neurons that have the same input and output, and *N*_L2/3 PC_ is the number of L2/3 PC.

## Results

### Effects of absence of LL-IN selectivity on single column under single stimulation

At the single neuron level, we use the aEIF model that is shown to reproduce different firing patterns^27,28^. The neuron model key parameters [membrane capacity (*C*_*m*_), leak conductance (*g*_*L*_), leak reversal potential (*E*_*l*_), reversal potential (*V*_*r*_), and threshold potential (*V*_*t*h_)] are estimated from the experimental literatures^8,9,29^. The mean values of all model parameters for different cell types are given in Table S1.

The single PFC column L2/3 PC are stimulated by applying triangular input patterns within 20 ms, and this response is considered as control (Fig. 1D). As our synaptic model adds STP, the network produces persistent activity after a short stimulus, which is considered to be one of the abilities to hold information^30,31^. For the next 160 ms (information retention period), the matrix is extracted and the accuracy of the information maintenance is calculated (see Section 2.4).

**Figure 2:**
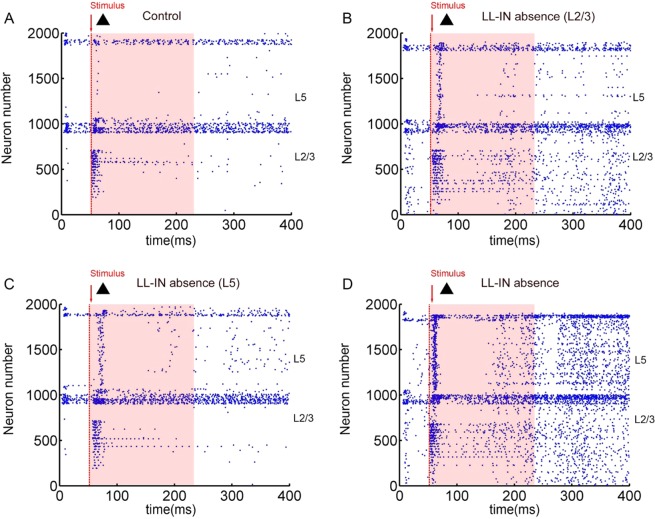
Spiking statistics of simulated PFC model networks. (**A**) Diagrams of PFC raster plots under stimulation. (**B**) Diagrams of L2/3 LL-IN absence raster plots of PFC under stimulation. (**C**) Diagrams of L5 LL-IN absence raster plots of PFC under stimulation. (**D**) Diagrams of (L2/3 + L5) LL-IN absence raster plots of PFC under stimulation. Red arrow and dotted line indicate that in 51 ms, triangular binary image starts to stimulate the L2/3 pyramidal cell. Shades of light red indicate the formation and maintenance of information.

Initially, ChCs are selectively absent, first in the supragranual layers 2/3 (Fig. 2B), then in the infragranular layer 5 (Fig. 2C), and finally in both the layers (Fig. 2D). It can be observed from the scatter diagram of spiking neurons that the effect of the absence of ChCs from the supragranual layers on the increase of network firing is more apparent than that of the infragranular layer interneurons.

**Figure 3:**
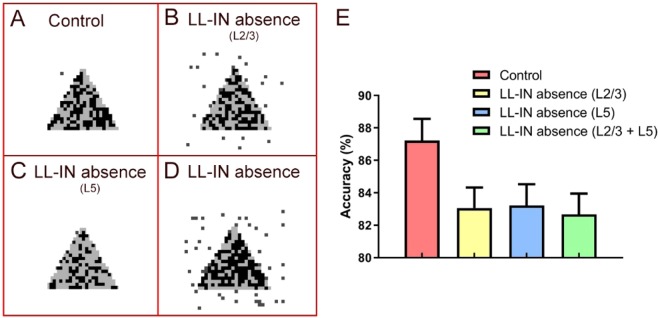
Comparison of completeness and accuracy in LL-IN absence under stimulation diagram. (**A**) Comparison of control input (gray) and output (dark). (**B**) Comparison of L2/3 LL-IN absence input (gray) and output (dark). (**C**) Comparison of L5 LL-IN absence input (gray) and output (dark). (**D**) Comparison of LL-IN absence input (gray) and output (dark). (**E**) Network memory maintenance accuracy, and the error bars represent the standard error of the mean of 15 independent experiments.

By reconstructing the output pattern, the effect of the absence of neurons on information maintenance becomes more intuitive. Compared to the control group, the loss of L5 ChCs has the least impact on information maintenance (Fig. 3A *vs* C), and the accuracy is 87.22 ± 2.34% *vs* 83.22 ± 1.31%. Since the ChCs are located mainly in the supragranular layer^32^, their absence significantly increases the noise of the output, decreasing the accuracy to 83.06 ± 1.27% (Fig. 3B). The total loss of all ChCs is likely to have a severe impact (Fig. 3D). More noise is recorded with an accuracy rate of only 82.67 ± 1.28% (Fig. 3E).

### Effects of absence of different types of interneurons on single column under single stimulation

**Figure 4:**
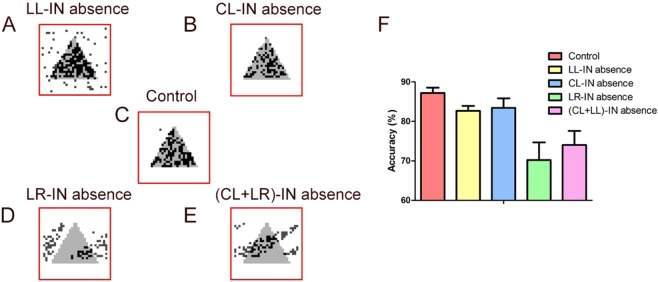
Comparison of completeness and accuracy in different types of interneurons under stimulation. (**A**) LL-IN absence input (gray) and output (dark) comparison. (**B**) CL-IN absence input (gray) and output (dark) comparison. (**C**) Control input (gray) and output (dark) comparison. (**D**) LR-IN absence input (gray) and output (dark) comparison. (**E**) (CL+ LR)-IN absence input (gray) and output (dark) comparison. **(F**) Network information maintenance accuracy; the error bars represent the standard error of the mean of 15 independent experiments.

The control, LL-IN, CL-IN, LR-IN, and (CL+ LR)-IN are absent in turn; the scatter diagram is shown in Figs. 2A and S1. The local inhibition is more important to stabilize the entire network in a single column than the long-range inhibition (Fig. 4A). The absence of LL-IN (Fig. S1A) accelerates the neuron spiking more apparently after stimulation than others (Fig. S1B–D). We speculate that the absence of CL-IN (Fig. 4B) has a weak effect on the network compared to the control (Fig. 4C) because of its proportion of only 4.1%, which is relatively small.

The long-range connections of interneurons affect the performance of the network differently. Without LR-IN, the output of the network is distorted (Fig. 4D), with accuracy as low as 70.22 ± 4.47% (Fig. 4F). When CL-IN and LR-IN are missing simultaneously, the output becomes more distorted (Fig. 4E), but the accuracy increasing slightly (74.00 ± 3.58%). Thus, the multi-angled, qualitative, and quantitative evaluations of network performance are indispensable.

### Effects of different types of supragranual layer interneurons on single column under multiple stimulations

**Figure 5:**
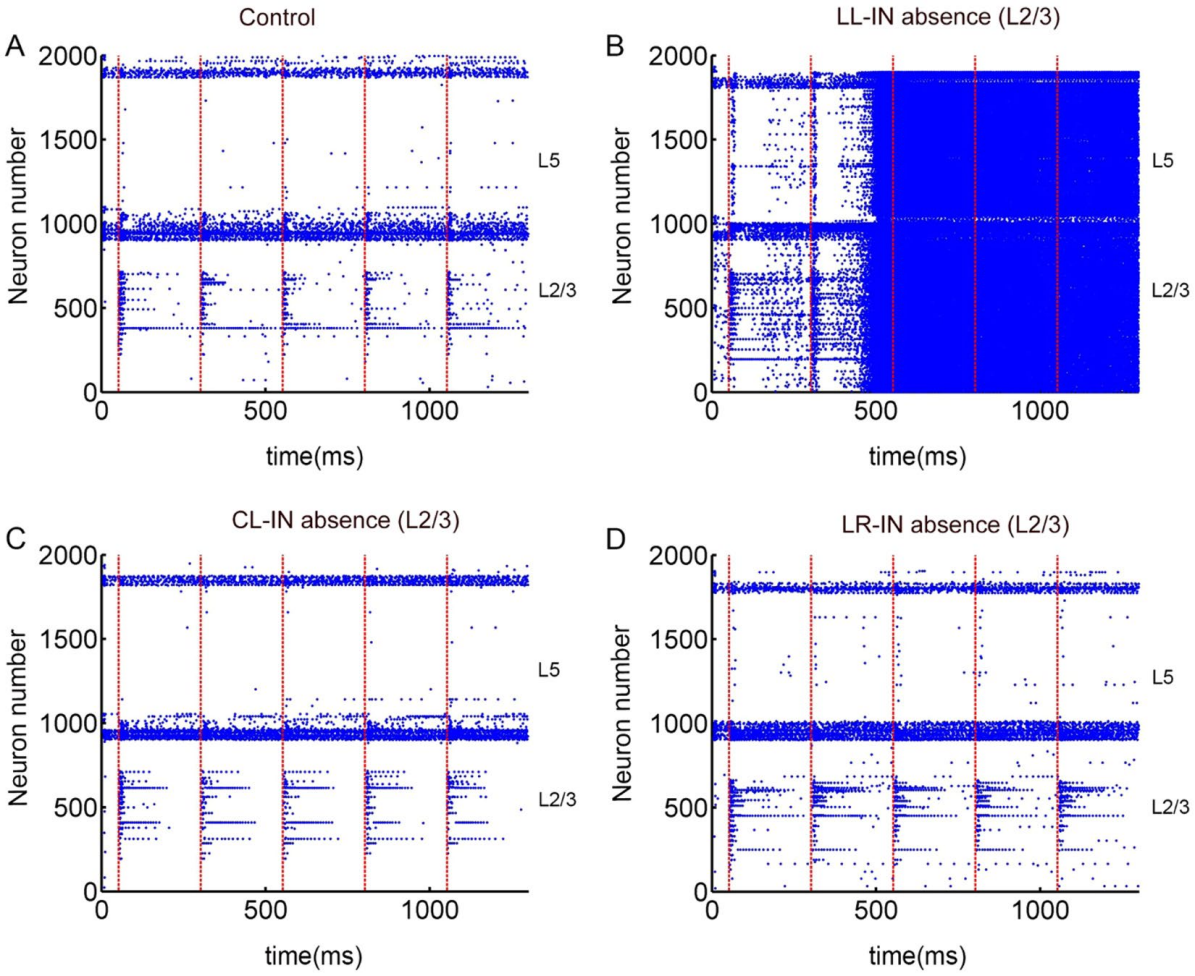
Spiking statistics of simulated PFC model networks under multiple stimulations. (**A**) The PFC raster plot under stimulation diagram. (**B**) The LL-IN absence raster plot of the PFC under stimulation diagram. (**C**) The CL-IN absence raster plot of the PFC under stimulation diagram. (**D**) The LR-IN absence raster plot of the PFC under stimulation diagram. Red dotted line indicates the moment of stimulus input of the L2/3 pyramidal cell.

In combination with the published literature, the layer 5 lacks DBCs, whereas the supragranual layer is relatively comprehensive in neuron types^8,13^. Hence, we choose five types of interneurons in the layer 2/3 as research objects, and let them remain missing in turn. During 51~70 ms, 301~320 ms, 551~570 ms, 801~820 ms, and 1051~1070 ms, the network is stimulated. The absence of LL-IN caused a disordered spiking compared with control after multiple stimulations (Fig. 5A,B). Even after a long period of stimulations, the network’s firing continues (Fig. S2). The entire network is highly excited, abnormally discharged, and produces symptoms similar to epilepsy. The absence of other types of interneurons has no significant effect on the single PFC column performance in the multi-stimulation diagram (Fig. 5C,D).

**Figure 6:**
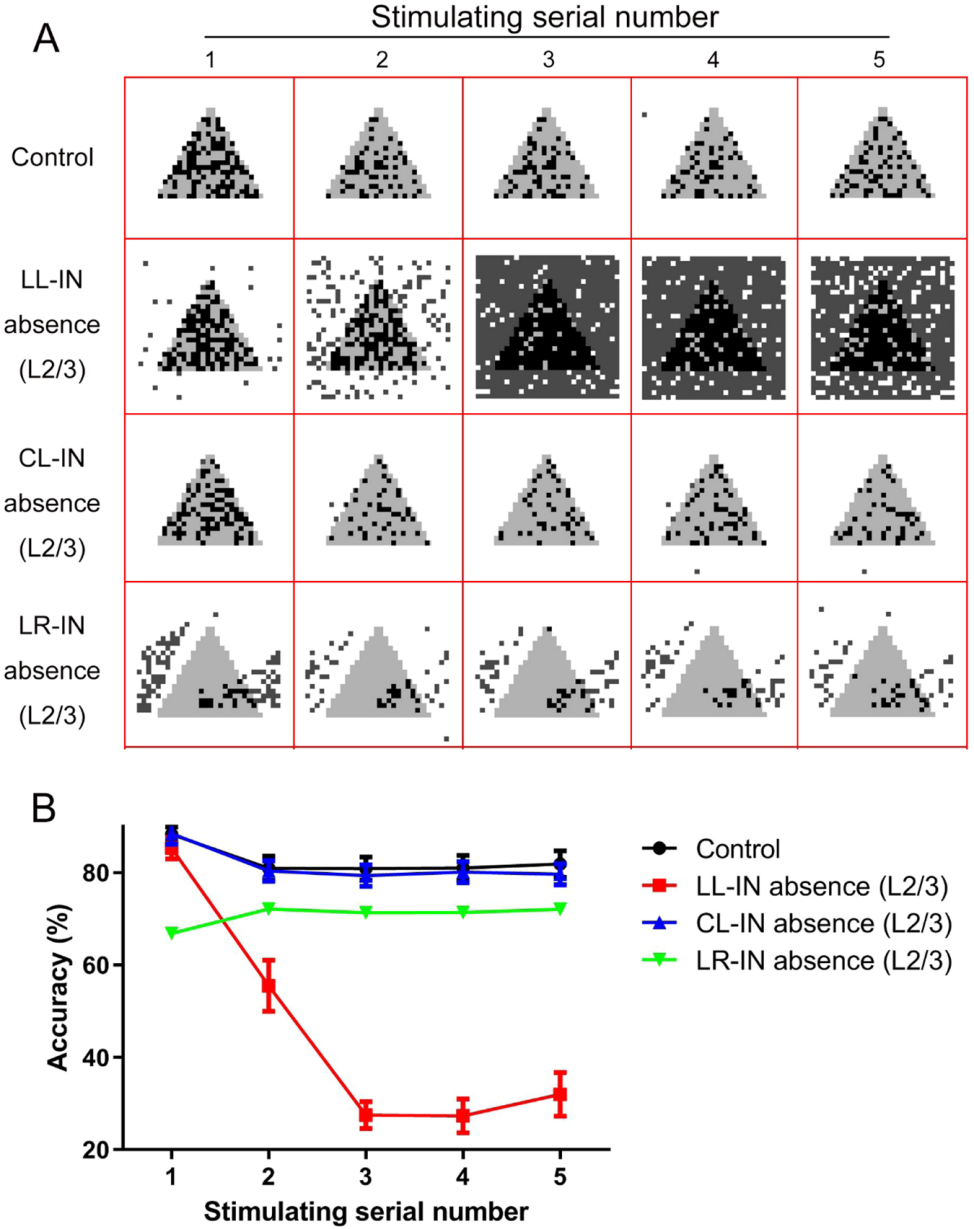
Comparison of completeness and accuracy of single column under multiple stimulations. (**A**) Control, LL-IN L2/3 absence, CL-IN L2/3 absence, LR-IN L2/3 absence input (gray) and output (dark) comparison under 5 times stimulation. (**B**) Network information maintenance accuracy under 5 times stimulation, the error bars represent the standard error of the mean of 15 independent experiments.

The output of the image reflects the performance of the network more intuitively. The local-layer inhibitive connection shows the greatest impact on the single column function, and the network has been unable to output images after the second stimulus. Consequently, the network cannot maintain all useful information (Fig. 6A). The accuracy rate, a quantitative indicator, directly reflects the extent to which the loss of different types of interneurons affects network function (Fig. 6B). The LL-IN absence accuracies are 85.28 ± 2.35%, 55.50 ± 5.57%, 27.50 ± 2.91%, 27.34 ± 3.62%, and 32.20 ± 4.73%. The absence of LR-IN distorts the output; however, it does not paralyze the network (Fig. 6A). The absence of CL-IN still has no significant effect on the single PFC column function. Its accuracy curve does not change significantly compared to that of the control group [Fig. 6B (red frame)].

### Role of multicolumn in maintaining network stability

**Figure 7:**
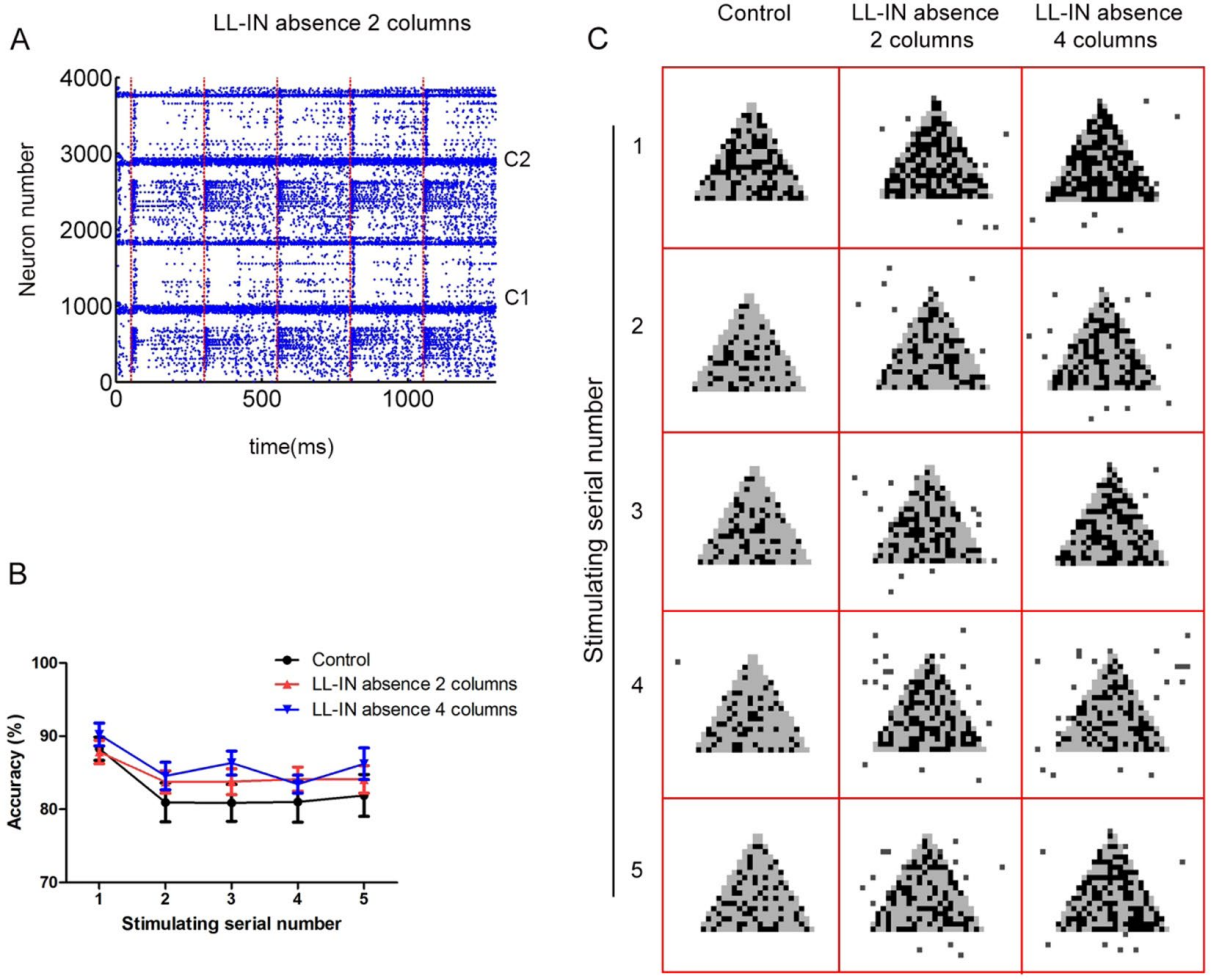
Completeness and accuracy in 2 columns and 4 columns PFC network under multiple stimulations. (**A**) The LL-IN L2/3 absence raster plot of 2 columns PFC under stimulation. Red dotted line indicates the moment of stimulus input of the layer 2/3 pyramidal cell. C1 and C2 mean column1and column2. (**B**) Network information maintenance accuracy under 5 times stimulation. The error bars represent the standard error of the mean of 15 independent experiments. (**C**) 2 columns and 4 columns LL-IN absence input (gray) and output (dark) comparison under 5 times stimulation.

The short-range connected interneurons are critical in maintaining the single column stability. We explore the interaction and cooperation between multiple columns in the absence of L2/3 LL-IN. When another column is added, the disorder spiking caused by the loss of local interneurons is greatly improved (Fig. 7A). Although both columns do not have short-range interneurons, the long-range inhibitory connections between the columns compensate for this lack of function. It is worth noting that the overall performance of two functional columns is even better than the control, which is reflected in the accuracy rate (Fig. 7B,C). The accuracies of five trials between LL-IN in the absence of 2 columns *vs* control are 89.56 ± 0.61% *vs* 90.11 ± 0.59%, 84.78 ± 0.53% *vs* 81.00 ± 0.67%, 84.44 ± 0.76% *vs* 80.56 ± 0.53%, 84.56 ± 0.63% *vs* 81.44 ± 0.77%, and 85.89 ± 0.86% *vs* 82.00 ± 0.87%. Furthermore, it is speculated that the long-range connection between the columns can compensate for the effects of the absence of subtype neurons, thus maintaining the network stability.

When the number of functional columns is increased to 4, even though the LL-IN is absent, the accuracy of the output after the second stimulation is higher than that of the control (Fig. 7B). It is clear that interneurons that have long-range projections compensate for the lack of local inhibition in the interaction between functional columns. This reconfirms the fact that although the functional columns of the new cortex are similar in structure, they are not arranged simply. The uniqueness of the connections between the functional columns shows that they can complement each other. The role of the long-range projection of interneurons is reflected in the multicolumn circuits.

## Discussion

We emphasize that the brain is modular, and each column of the neocortex is analogous to a local hub. Although the structures of the columns are similar, the various connections between them render the columns critical in high-level cognitive functions. The premise of diversified connections is the various types of interneurons. The different types of interneurons seem to specifically inhibit the aspects of cortical circuit operation, such as balance excitation, regulation gain, and generating oscillations^33–35^. Our research focuses on the division of inhibitory mechanisms for the maintenance of information in multicolumn PFC network.

The five types of interneurons in our model are appropriately interpreted according to their extending range of axonal projections. For instance, the ChCs as an important member of LL-IN type in the parvalbumin interneuron family is not negligible^36^. Some long-range connection interneurons have a huge axonal cluster that extends not only to the cross-layers, but also to cross-columns, and even multiple columns. Based on this fact, we use the conventional BPCs and DBCs to represent CL-IN. LBC and MC are two main compositions of LR-IN^8,12^. Classification of neurons based on morphology, protein expression and synaptic characteristics has certain limitations. All we can do is minimize types overlap.

Previously published literature has revealed that the ChCs not only mediate the directional inhibitory control between local PC ensembles, but may also shape the communication hierarchy and route information flow between global networks^36^. This is consistent with our simulation result that LL-IN represented by ChCs is crucial for maintaining network stability. If ChCs are absent, the network will not be able to transmit information effectively (Figs. 3, 4A and 5B). Without the local suppression, the network will be disordered and will not function normally. On the other hand, the effect of the absence of LR-IN on the network is reflected in the severe deformation of the output image. From the aspect of computational modeling, it is proved that different connection lengths lead to different divisions of labor in information maintenance.

Recently, there have been many studies on the computational modeling of large-scale circuits because many characteristics are difficult to be represented in local networks, such as network hierarchy and signal propagation^37,38^. We have also made a preliminary attempt in the model. In the multicolumn structure, a long-range projection of interneurons is essential when local interneurons are missing. The cross-column inhibition connection can replace the local connection and maintain the ability to actively hold information for a long time (Fig. 7).

Although the anatomical fine structure of the neocortex is uniform, the columns do not simply repeat, but functionally interact with each other. This model provides a tool to investigate the cortical organization. The redundant design of the brain improves the stability of the nervous system. This may explain why some patients undergo a small partial resection of the cortical brain tissue, while the basic function remains unaffected.

## Supplementary information

Supplementary Information.

## Data Availability

All data generated or analyzed during this study are included in this published article (and its Supplementary Information files).
