# Supplementary material for: Computational Investigation of Contributions from Different Subtypes of Interneurons in Prefrontal Cortex for Information Maintenance
Source: Sci Rep. 2020 Mar 13;10:4671. doi: 10.1038/s41598-020-61647-2 (PMC7070096; doi:10.1038/s41598-020-61647-2)
Supplement: Supplementary file 1 — Supplementary Information. [file 41598_2020_61647_MOESM1_ESM.pdf]

Supplementary Information for

Computational Investigation of Contributions from Different Subtypes of Interneurons in Prefrontal Cortex for Information Maintenance

Qian Zhang[1, 2,*, +], Yi Zeng[1, 2, 3, 4, *, +], and Taoyi Yang[1]

[1] Research Center for Brain-inspired Intelligence, Institute of Automation, Chinese Academy of Sciences, Beijing, 100190, China.

[2] University of Chinese Academy of Sciences, Beijing, 100049, China.

[3] Center for Excellence in Brain Science and Intelligence Technology, Chinese Academy of Sciences, Shanghai, 200031, China

[4] National Laboratory of Pattern Recognition, Institute of Automation, Chinese Academy of Sciences, Beijing, 100190, China

[*] **Correspondence:**
Qian Zhang and Yi Zeng
q.zhang@ia.ac.cn and yi.zeng@ia.ac.cn

[+] These authors contributed equally.

Figures

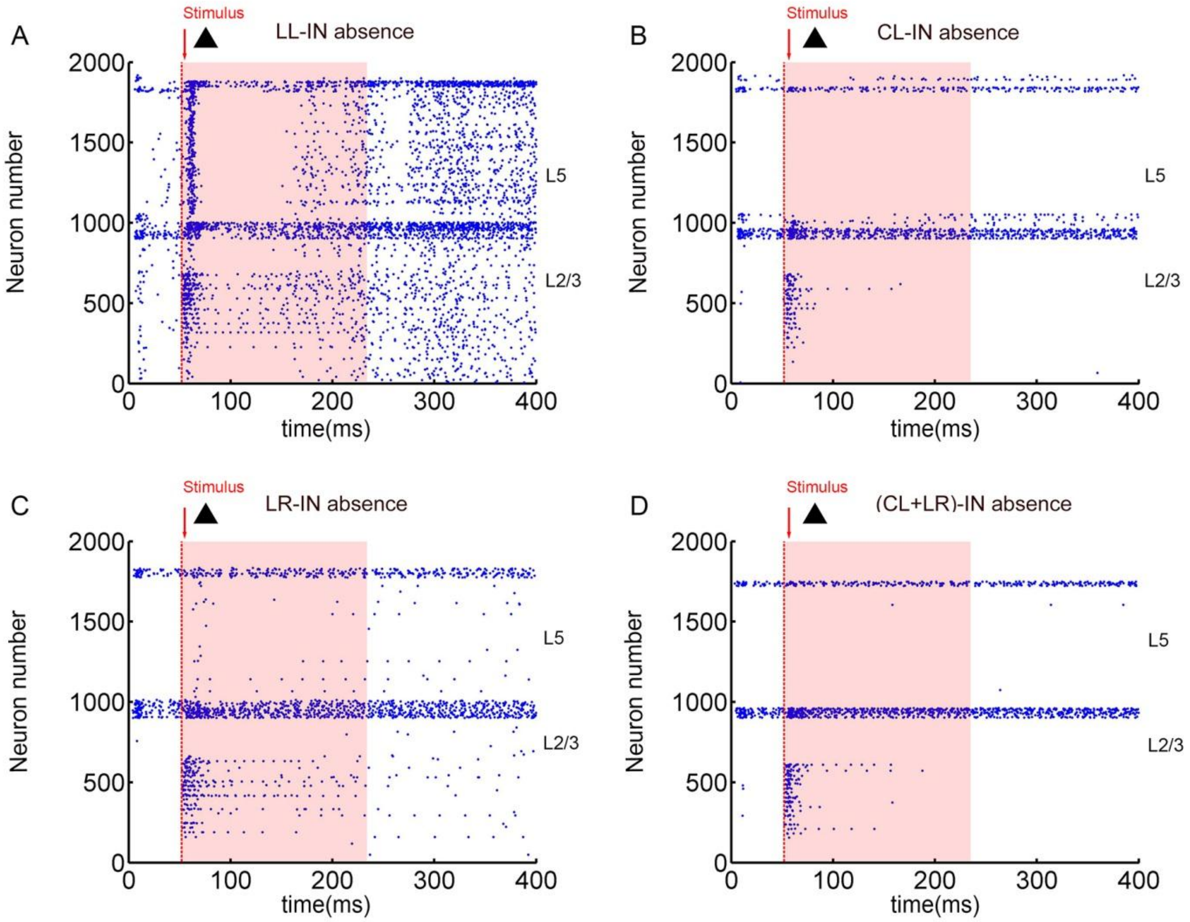

Figure S1. Spiking statistics of simulated PFC model networks. (A)-(D) The LL-IN absence, CL-IN absence , LR-IN absence and (CL+LR)-IN raster plot of the PFC under stimulation diagram. The red arrow and dotted line indicate that in 51ms, triangular binary image stars to stimulate the L2/3 pyramidal cell. The shades of light red indicate the formation and maintenance of information.

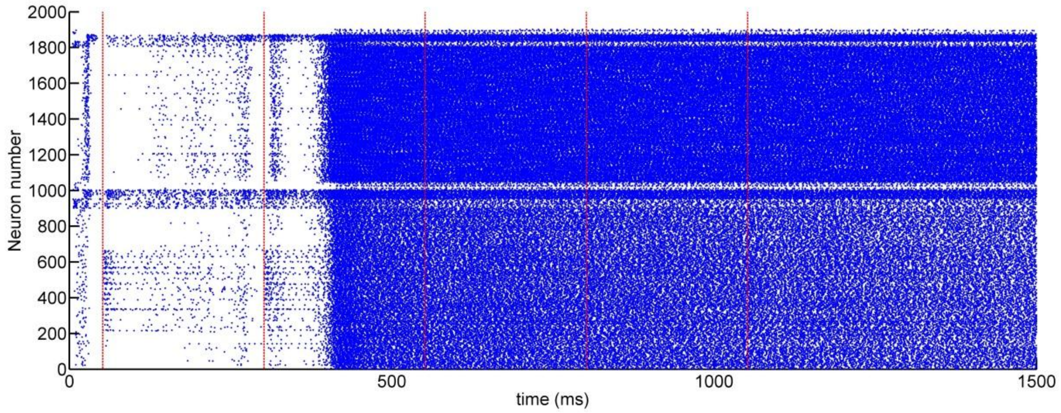

Figure S2. Spiking statistics of L2/3 LL-IN absence networks under multi stimulation. Red dotted line indicates the moment of stimulus input the L2/3 pyramidal cell.

Tables

TABLE S1

Neuron model parameter

	L2 /3					L5			
	PC	LL-IN (ChC)	CL-IN-a (BPC)	CL-IN-b (DBC)	LR-IN-b (MC)	PC	LL-IN (ChC)	CL-IN-a (BPC)	LR-IN-b (MC)
C_m(pF)	165.4	59.4	10.0	15.0	86.3	241.5	53.8	10.0	82.9
g_L(nS)	7.2	5.3	3.9	1.5	2.9	7.4	4.9	3.4	3.0
E_L(mV)	-85.0	-85.2	-84.9	-76.0	-70.6	-80.6	-85.2	-76.8	-72.8
V_r(mV)	-110.9	-71.3	-77.4	-67.6	-66.3	-67.8	-71.3	-77.4	-66.6
V_{th}(mV)	-52.1	-43.8	-46.4	-41.9	-42.4	-48.6	-43.8	-46.4	-48.2
b(pA)	7.2	34.9	5.6	3.0	3.7	8.3	34.9	5.6	4.2
τ_W (ms)	23.1	4.4	4.2	4.6	9.1	33.2	4.4	4.2	9.1
a(nS)	2.0								
$\triangle_T$(mv)	20.0								

Table S1. Neuron model parameter. The LBC have similar electrophysiological properties to the PCs in the respective layers. C_m is the membrane capacitance, g_L is the leak conductance, E_L is the leak reversal potential, V_{th} is the spike threshold, Δ_T is the slope factor, τ_W is the adaption time constant, *a* is the subthreshold adaptation and *b* is the spike-triggered adaptation. PC: pyramidal cell, LL-IN: local-layer connection interneuron, CL-IN: cross-layer connection interneuron, CC-IN: cross-column connection interneuron, LR-IN: long-range connection interneuron. ChC: chandelier cell, BPC: bipolar cell, DBC: double-bouquet cell and MC: Martinotti cell.

TABLE S2

Related to synapses parameters

	**$GABA_A$**	**AMPA**	**NMDA**
E_{rev}(mV)	-70	0	0
τ_{on}(ms)	3	1.4	4.3
τ_{off}(ms)	40	10	75

Table S2. Related to synapses parameters. E_{rev} is the reversal potential, τ_{off} and τ_{on} are the onset and the offset time constants.

TABLE S3

Related to connection parameters

pre	post	p_{con}	g_{max}	τ_D(ms)
PC L2/3	PC L2/3	0.139	0.84	1.55
PC L2/3	PC L5	0.233	0.95	1.91
PC L5	PC L2/3	0.045	0.84	2.75
PC L5	PC L5	0.081	0.88	1.56
PC L2/3	LL-IN L2/3	0.325	1.34	0.96
PC L2/3	CL-IN-a L2/3	0.159	0.47	0.96
PC L2/3	LR-IN-a L2/3	0.325	1.34	0.96
PC L2/3	LR-IN-b L2/3	0.290	0.25	0.96
PC L2/3	LL-IN L5	0.087	0.77	1.18
PC L2/3	CL-IN-a L5	0.080	0.27	1.18
PC L2/3	LR-IN-a L5	0.087	0.77	1.18
PC L2/3	LR-IN-b L5	0.150	0.14	1.18
PC L5	LL-IN L2/3	0.188	1.52	1.05
PC L5	CL-IN L2/3	0.092	0.53	1.05
PC L5	LR-IN-a L2/3	0.188	1.52	1.05
PC L5	LR-IN-b L2/3	0.168	0.28	1.05
PC L5	LL-IN L5	0.333	2.3	0.60
PC L5	CL-IN-a L5	0.080	0.13	0.60
PC L5	LR-IN-a L5	0.333	2.3	0.60
PC L5	LR-IN-b L5	0.362	1.91	0.60
LL-IN L2/3	PC L2/3	0.466	2.30	1.25
CL-IN-a L2/3	PC L2/3	0.301	0.13	1.25
LR-IN-a L2/3	PC L2/3	0.466	2.30	1.25
LR-IN-b L2/3	PC L2/3	0.710	1.91	1.25
LL-IN L2/3	PC L5	0.217	1.07	1.54
CL-IN-a L2/3	PC L5	0.140	0.06	1.54
LR-IN-a L2/3	PC L5	0.217	1.07	1.54
LR-IN-b L2/3	PC L5	0.330	0.89	1.54
LL-IN L5	PC L2/3	0.039	0.1	1.44
CL-IN-a L5	PC L2/3	0.027	0.04	1.44
LR-IN-a L5	PC L2/3	0.039	0.1	1.44
LR-IN-b L5	PC L2/3	0.040	0.07	1.44
LL-IN L5	PC L5	0.274	0.69	0.82
CL-IN-a L5	PC L5	0.173	0.3	0.82
LR-IN-a L5	PC L5	0.274	0.69	0.82
LR-IN-b L5	PC L5	0.282	0.5	0.82

Table S3. Related to connection parameters. The connection parameters of CL-IN-b L2/3 are the same as those of CL-IN-a L2/3. Mean of the synapses connection between pre- and post-synaptic pair neurons, p_{con} is connection probability, g_{max} is peak conductance, τ_D is transmission delay.
